# Change of right ventricular systolic pressure can indicate dasatinib‐induced pulmonary arterial hypertension in chronic myeloid leukemia

**DOI:** 10.1002/cam4.3588

**Published:** 2021-02-15

**Authors:** Sung‐Eun Lee, Jee Hyun Kong, Soo‐Hyun Kim, Eun‐Jung Jang, Nack‐Gyun Chung, Bin Cho, Suk Joong Oh, Hae‐Eok Jung, Ho‐Joong Youn, Woo‐Baek Chung, Dong‐Wook Kim

**Affiliations:** ^1^ Department of Hematology Catholic Hematology Hospital College of Medicine The Catholic University of Korea Seoul South Korea; ^2^ Division of Hematology Department of Internal Medicine Wonju Christian Hospital Yonsei University College of Medicine Wonju South Korea; ^3^ Leukemia Research Institute The Catholic University of Korea Seoul South Korea; ^4^ Department of Pediatrics Catholic Hematology Hospital College of Medicine The Catholic University of Korea Seoul South Korea; ^5^ Department of Hematology Kangbuk Samsung Hospital Sungkyunkwan University School of Medicine Seoul South Korea; ^6^ Division of Cardiology College of Medicine The Catholic University of Korea Seoul South Korea

**Keywords:** chronic myeloid leukemia, dasatinib, echocardiography, pulmonary arterial hypertension, right ventricular systolic pressure

## Abstract

**Background:**

We investigated the feasibility of the clinical application of non‐invasive transthoracic echocardiography for diagnosis of pulmonary arterial hypertension induced by dasatinib (D‐PAH) in chronic myeloid leukemia (CML).

**Methods:**

A total of 451 CML patients who were examined by 2D‐echocardiography at least once at baseline and/or during dasatinib therapy as frontline (*n* = 196) and subsequent line (*n* = 255) therapies were included in this study. D‐PAH was defined as right ventricular systolic pressure (RVSP) >40 mm Hg with relevant symptoms and the absence of other specific etiologies.

**Results:**

A total of 847 echocardiographies were performed including at baseline (*n* = 255) and during dasatinib treatment (*n* = 592). During the median of 36.2 (0.1–181.8) months of dasatinib therapy, the level of RVSP gradually increased (Spearman's *r* = 0.2819, *p* < 0.001) and the mean RVSP was significantly increased after taking dasatinib therapy compared with baseline. During dasatinib therapy, 56 (12.4%) patients had RVSP >40 mm Hg without (asymptomatic, *n* = 27, 48.2%) or with symptoms (D‐PAH, *n* = 29, 51.8%). All asymptomatic patients maintained dasatinib therapy without further symptoms and the D‐PAH patients ultimately switched to other tyrosine kinase inhibitors. After dasatinib discontinuation, 13 (45%) and 15 (52%) patients showed RVSP normalization and gradual decrease, respectively.

**Conclusions:**

Our large cohort study demonstrated that the gradual increment of RVSP might be induced by dasatinib and non‐invasive echocardiography can be fast way for early diagnosis as well as for monitoring of D‐PAH.

## Graphical abstract

(a) The aim of this study was to investigate the fidelity of 2D‐echocardiography for diagnosis of pulmonary arterial hypertension induced by dasatinib (D‐PAH) in chronic myeloid leukemia. (b) The gradual increment of right ventricular systolic pressure might be induced by dasatinib and non‐invasive echocardiography can be fast way for early diagnosis as well as for monitoring of D‐PAH.

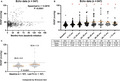

## 1 INTRODUCTION

Pulmonary arterial hypertension (PAH) is a rare disorder characterized by progressive obliteration of the pulmonary microvasculature resulting in elevated pulmonary vascular resistance and premature death.^1^ PAH can be idiopathic, heritable, or caused by drugs and has been associated with connective tissue diseases, human immunodeficiency syndrome, and portal hypertension.^2^ Dasatinib has been reported to be one of the causes of drug‐induced PAH, with an estimated incidence of 0.45%.^3^, ^4^, ^5^, ^6^, ^7^, ^8^, ^9^ Among confirmed dasatinib‐induced PAH (D‐PAH) patients, approximately 60% completely resolve with permanent discontinuation of treatment; however, most patients show severe functional and hemodynamic signs, and some need intensive unit care management.^10^, ^11^ The primary mechanism of D‐PAH has been suggested as pulmonary endothelial damage via increased mitochondrial ROS production by chronic dasatinib therapy.^12^

Given the long life expectancy of chronic phase chronic myeloid leukemia (CP CML) patients in the era of tyrosine kinase inhibitors (TKIs),^13^ minimizing the toxicity of TKIs and maintaining quality of life are becoming more important in long‐lasting treatment. Thus, detection of D‐PAH and its relevant risk factors might be important. However, a definitive diagnosis of PAH requires right heart catheterization (RHC),^14^ which is invasive procedure that is associated with morbidity (1.1%) and mortality (0.055%) even when performed at experienced centers.^15^ This might be a reason for the potential under‐reporting D‐PAH and for the requirement of readily applying measurement.^10^

With the introduction of the transthoracic echocardiography, approximate evaluation of pulmonary arterial pressure (PAP) became feasible.^16^ Although echocardiograph has high false‐positive rates,^17^, ^18^ it is non‐invasive, readily available, and relatively inexpensive compared with RHC.^19^ Currently, right ventricular systolic pressure (RVSP) is recommended not only as a screening tool for PAH but also as a method of monitoring in patients with relevant symptoms and signs.^16^, ^20^ However, as the level of RVSP can be influenced by several factors such as congenital heart disease, pleural effusion, and pericardial effusion,^1^, ^8^, ^12^ the diagnosis of D‐PAH by RVSP elevation should be made with caution in patients with pleural effusion or pericardial effusion, which frequently develop on dasatinib treatment.

Here, we investigated the fidelity of 2D‐echocardiography for the evaluation of D‐PAH according to change of RVSP by chronic dasatinib therapy for CML. We also analyzed the clinical characteristics of D‐PAH.

## 2 METHODS

### 2.1 Patients

Among the 679 patients who were diagnosed with CP CML and treated with first‐line dasatinib and second‐line or more from March 2005 to December 2018, we included 451 patients who were examined by 2D‐echocardiography examination at least once at pre‐treatment of dasatinib and/or during dasatinib therapy. Among 451 patients, 156 patients received dasatinib in clinical trials and 295 patients were treated in clinical practice. Evaluation with 2D‐echocardiography was performed for cardiovascular disease screening and/or when patients had dyspnea and chest discomfort after dasatinib initiation. For the differential diagnosis of pulmonary hypertension, chest x‐ray, electrocardiogram (ECG), RHC, chest computed tomography angiogram (angio‐CT), and laboratory tests for D‐dimer, brain natriuretic peptide (BNP), autoimmune disease, and human immunodeficiency virus (HIV) were performed. Echocardiography monitoring was employed until normalization of RVSP with resolution of clinical manifestations after treatment modification. All echocardiography results were carefully assessed by designated cardiologists. This study was approved by the institutional review board of Seoul St. Mary's Hospital, The Catholic University of Korea and conducted in accordance with the Declaration of Helsinki.

### 2.2 Dasatinib treatment

Dasatinib was initiated at ≤100 mg daily for CP and ≥140 mg daily for advanced phases. Hematologic, cytogenetic, and molecular responses were evaluated regularly. To assess cytogenetic response, a minimum of 20 metaphases was examined in bone marrow samples. For evaluation of molecular response, duplicate qRT‐PCR and nested RT‐PCR with at least 4.5‐log sensitivity was performed in our laboratory (Leukemia Research Institute, The Catholic University of Korea, Seoul, Korea). In cases of hematologic and nonhematologic adverse events (AEs), the dasatinib dose was reduced or was transiently discontinued. AEs were continuously assessed and graded according to the National Cancer Institute's Common Terminology Criteria for Adverse Event version 4.0.

### 2.3 Definition and treatment of D‐PAH

According to the guidelines of the American Heart Association (AHA), D‐PAH was defined as RVSP >40 mm Hg on echocardiography with relevant symptoms and the absence of other specific etiologies known to evoke PAH or RVSP change such as coronary artery disease, pulmonary thromboembolism, congenital heart disease, connective tissue disease, left heart disease, or the effects of other drugs or toxins except dasatinib. RVSP was estimated by 4× (tricuspid regurgitant jet maximum velocity)^2^ + right atrial pressure. Right atrial pressure was estimated according to 2010 American Society of Echocardiography (ASE) Guidelines for Echocardiographic Assessment of Right Heart in Adult,^21^ and inferior vena cava diameter was measured according to 2015 ASE Recommendations for Cardiac Chamber Quantification by Echocardiography in Adults.^22^ In cases of concomitant AEs such as pleural effusion or pericardial effusion, RVSP levels were repeatedly evaluated until the events was resolved by chest x‐ray or echocardiography. After development of D‐PAH, we carefully monitored the improvement of hemodynamic profiles including clinical symptoms after discontinuation or dose reduction of dasatinib. Sildenafil, bosentan, iloprost, diuretics, and/or calcium channel blockers were administered concurrently as required.

### 2.4 Statistical analysis

Statistical comparisons between groups were performed using the Mann‐Whitney test and Wilcoxon test for continuous variables. Chi‐square test and Fisher's exact test were applied for categorical variables. Spearman's rho was used for correlation. The cumulative incidence of D‐PAH was calculated as time from dasatinib initiation to D‐PAH diagnosis or last follow‐up by Kaplan–Meier method. A *p* < 0.05 was considered statistically significant and all *p* values correspond to two‐sided significance tests.

## 3 RESULTS

### 3.1 Patient characteristics and echocardiography data collection

The patient characteristics are summarized in Table 1. The median age at dasatinib initiation was 46 (7–81) years and 59.4% were male. Prior to dasatinib, interferon and allogeneic hematopoietic stem cell transplantation were performed in 30 (6.7%) and 5 (1.1%) patients, respectively. A total of 196 patients (43.5%) received first‐line dasatinib therapy and 255 (56.5%) patients received dasatinib as a subsequent line therapy after prior TKI. Dasatinib was started a median of 7.3 months (0–348.4) after CML diagnosis and was administered for a median of 36.2 months (0.1–181.8) with median mean daily dose of 85 mg (36–151). During dasatinib treatment, pleural effusion developed in 215 patients (47.7%).

**Table 1:** Characteristics of patients

	*n* = 451
Age at CML diagnosis, years, median (range)	46 (7–81)
Age at dasatinib initiation, years, median (range)	49 (15–81)
Sex, male, *n* (%)	268 (59.4)
Disease phase at dasatinib initiation	
CP/AP/BP	420 (93.1)/8 (1.8)/23 (5.1)
Dasatinib treatment, *n* (%)	
Frontline	196 (43.5)
Second line	162 (35.9)
Beyond second line	93 (20.6)
Dasatinib initial dose, mg/day	
≤100	356 (79)
≥140	95 (21)
Previous treatment	
Interferon, *n* (%)	30 (6.7)
Allogeneic HSCT, *n* (%)	5 (1.1)
Prior TKI before dasatinib	
Imatinib, *n* (%)	231 (51.2)
Nilotinib, *n* (%)	41 (9.1)
Bosutinib, *n* (%)	10 (2.2)
Radotinib, *n* (%)	70 (15.5)
Ponatinib, *n* (%)	1 (0.2)
Time from CML diagnosis to dasatinib initiation, months, median (range)	7.3 (0–348.4)
Dasatinib treatment duration, months, median (range)	36.2 (0.1–181.8)
Mean daily dose of dasatinib, mg/day, median (range)	85 (36–151)
Pleural effusion, *n* (%)	215 (47.7)
Echocardiography	847
Frequency of echocardiography, median (range)	2 (1–7)
Patients who received echocardiography once, *n* (%)	204 (45.2)
Patients who received echocardiography at least two times, *n* (%)	247 (54.8)
Median interval of echocardiography, months (range)	11.2 (0.1–103.1)

Abbreviations: CML, Chronic Myeloid Leukemia; HSCT, Hematopoietic Stem Cell Transplantation; TKI, Tyrosine Kinase Inhibitor.

A total of 847 echocardiography evaluations were performed in 451 patients for a median of 2 times (range 1–7) at baseline (pre‐treatment of dasatinib) and/or during dasatinib therapy (Table 1). More than half of patients (*n* = 247, 55%) received echocardiography examination at least two times with a median interval of 11.2 months (0.1–103.1), and 204 patients (45%) were only evaluated once.

### 3.2 RVSP change during dasatinib therapy

**Figure 1:**
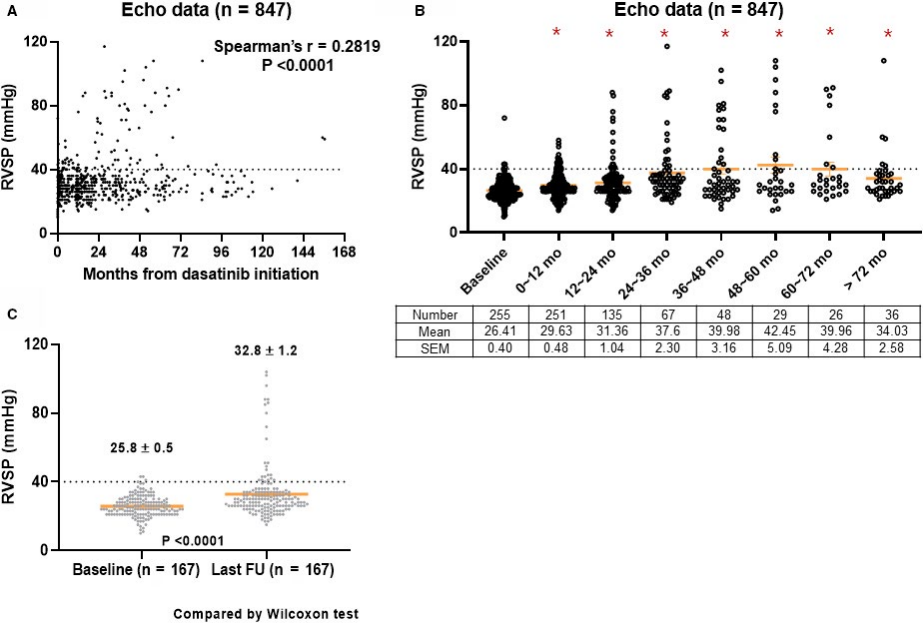
Right ventricular systolic pressure (RVSP) change during dasatinib therapy. A total of 847 RVSP data from echocardiography during dasatinib therapy was plotted according to the implemented time from dasatinib initiation (A). RVSP values were plotted in 1‐year intervals (B). For patients who had paired baseline RVSP and at least one RVSP during dasatinib therapy, baseline RVSP was compared to the last available RVSP during dasatinib therapy (C). *p < 0.05 compared with baseline

First, we plotted all RVSP data according to the implemented time from dasatinib initiation to investigate the correlation between RVSP and dasatinib treatment duration (Figure 1A). A total of 847 RVSP data measured during dasatinib therapy was positively correlated with the implemented time from dasatinib initiation (Spearman's *r* = 0.2819, *p* < 0.001). Next, we compared RSVP according to 1‐year intervals: RVSP measured at baseline (*n* = 255) and during dasatinib treatment (251, 135, 67, 48, 29, 26, and 36 echocardiography data during 0–1, 1–2, 2–3, 3–4, 4–5, 6–7, and >7 years) (Figure 1B). At baseline, the mean RVSP was 26.41 ± 0.4 mm Hg, which significantly increased to 29.63 ± 0.48 mm Hg (*p* < 0.001), 31.36 ± 1.04 mm Hg (*p* < 0.001), 37.6 ± 2.3 mm Hg (*p* < 0.001), 39.98 ± 3.16 mm Hg (*p* < 0.001), 42.45 ± 5.09 mm Hg (*p* < 0.001), and 39.96 ± 4.28 mm Hg (*p* < 0.001) at 0–1, 1–2, 2–3, 3–4, 4–5, and 6–7 years of dasatinib therapy, respectively.

A total of 167 patients had serial RVSP data at baseline and during dasatinib therapy. The comparison of RVSP data on paired baseline and last available data on dasatinib is shown in Figure 1C (25.8 ± 0.5 vs. 32.8 ± 1.2 mm Hg, *p* < 0.001 by Wilcoxon test).

### 3.3 RVSP change according to mean daily dose of dasatinib

**Figure 2:**
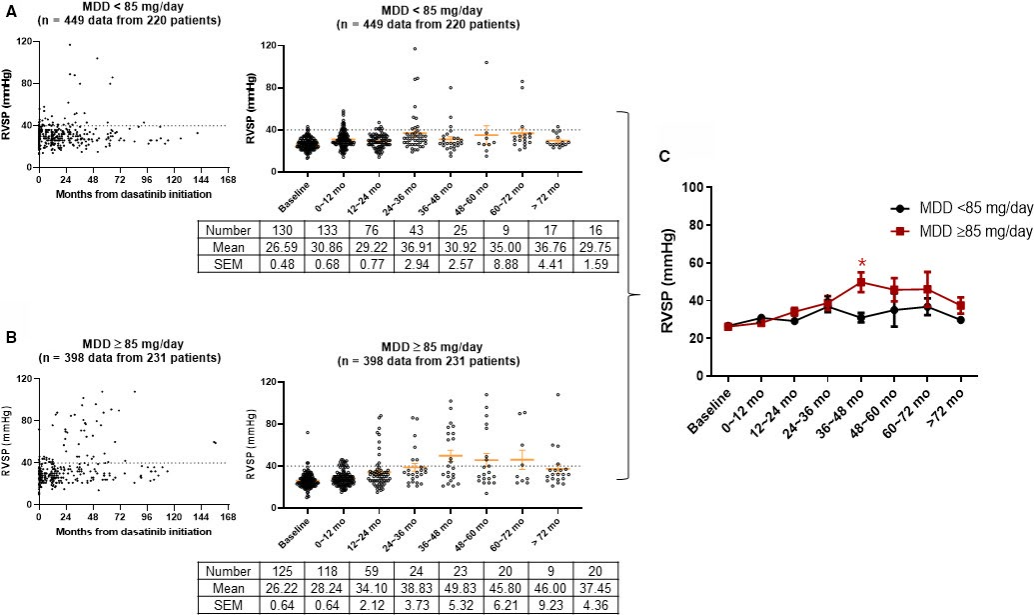
Right ventricular systolic pressure (RVSP) change according to mean daily dose of dasatinib. The patients were grouped into low (<85 mg/day; *n* = 220) and high (≥85 mg/day; *n* = 231) mean daily dose groups. The RVSP of both groups was plotted according to implemented time and 1‐year interval from dasatinib initiation (A, B). RVSP changes in both groups were compared (C)

To evaluate the effect of dasatinib dose on RVSP change during dasatinib therapy, we grouped the patients into low (<85 mg/day; *n* = 220) and high (≥85 mg/day; *n* = 231) mean daily dose groups by the value chosen to maximize the difference between the two groups. We plotted RVSP according to the implemented time and 1‐year interval from dasatinib initiation (Figure 2A, B). We compared RVSP change between low and high dose groups (Figure 2C). The trend of RVSP increasing over time was observed more clearly in the high‐dose group, and the mean RVSPs at 3–4 years (*p* = 0.032) were significantly higher in the high‐dose group than the low dose group. Additionally, RVSP changes according to initial dose of dasatinib (≤100 mg and ≥140 mg) were analyzed, which showed a trend for more highly increasing RVSP in high initial dose group than the low initial dose group (data no shown).

In addition, we compared RVSP change between frontline and subsequent line dasatinib treatment groups (Figure S1).

### 3.4 Characteristics of patients with RVSP over 40 mm Hg

Among the 56 patients who had RVSP >40 mm Hg during dasatinib therapy, 29 (51.8%) patients were diagnosed as D‐PAH with relevant clinical manifestations. The other 27 patients were ruled out for D‐PAH; 26 patients were asymptomatic and one patient was diagnosed as non‐ST elevation myocardial infarction (NSTEMI) confirmed by coronary angiography. Thus, these 27 patients were grouped into the asymptomatic group for whom dasatinib therapy was maintained without further symptoms. Among them, 13 patients were evaluated with follow‐up echocardiography since the date of RVSP >40 mm Hg; all results were within normal limits (*n* = 8) or did not show a change or decrease (*n* = 4), except for one patient who was diagnosed as NSTEMI and received percutaneous coronary intervention (Figure S2). The other 14 patients maintained dasatinib therapy without further symptoms by the cutoff date and follow‐up echocardiograpy was in planning.

We compared the clinical characteristics of all patients with RVSP >40 mm Hg by evaluating the D‐PAH group (*n* = 29) versus asymptomatic group (*n* = 27; Table S1). The mean value of the highest RVSP was higher in the D‐PAH group compared with the asymptomatic group (79.4 ± 4.0 vs. 44.0 ± 0.8 mm Hg, *p* < 0.001). The duration from dasatinib initiation to RVSP >40 mm Hg were significantly different in D‐PAH and asymptomatic groups, with 39.2 (range, 16.1–155.2) and 8.8 (range, 0.3–92.9) months, respectively (*p* < 0.001). The median age at baseline was higher in the asymptomatic group than the D‐PAH group (61 years [range, 26–75] vs. 46 years [range, 21–70], *p* < 0.001). Other clinical variables, including previous treatment (interferon, allogeneic HSCT, or prior TKI to dasatinib), were not significantly different between two groups.

### 3.5 Incidence of dasatinib‐induced PAH

**Figure 3:**
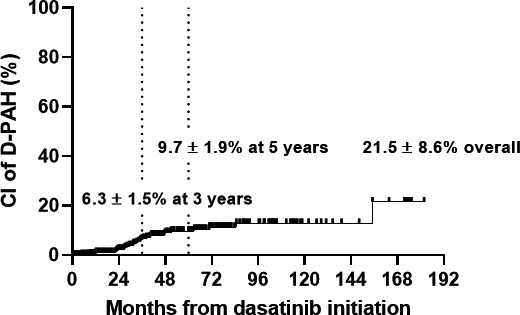
Cumulative incidence of clinical dasatinib‐induced PAH (D‐PAH). The estimated cumulative incidences of D‐PAH at 3‐ and 5‐years were 6.3 ± 1.5% and 9.7 ± 1.9%, respectively

Out of 451 patients, 29 patients (6.4%) were finally defined as D‐PAH. Their clinical characteristics are listed in Table 2. The median age at the time of D‐PAH diagnosis was 48 years (27–72), and 15 patients (52%) were female. The estimated cumulative incidences of D‐PAH at 3‐ and 5‐years were 6.3 ± 1.5% and 9.7 ± 1.9%, respectively (Figure 3). Dasatinib was used as frontline treatment in 9 patients (31%). Seven patients (24%) received interferon and one received allogeneic HSCT before dasatinib. Pleural effusion existed in 22 (76%) patients prior to or concurrently with D‐PAH. Seven patients received RHC to confirm D‐PAH. Additional chest CT angiography was performed in three patients and showed compatible finding with pulmonary hypertension.

**Table 2:** Characteristics of patients with dasatinib‐induced pulmonary arterial hypertension

Patient	Age at D‐PAH diagnosis (year)	Sex	Time from CML diagnosis to DAS initiation (months)	Previous therapy	Pleural effusion	DAS treatment duration before D‐PAH (months)	RVSP at D‐PAH diagnosis (mm Hg)	LHD and LVEF(%) at D‐PAH diagnosis	The highest RVSP (mm Hg)	The latest RVSP (mm Hg)	DAS treatment duration after D‐PAH (months)	Treatment of D‐PAH	Switch to other TKI	Additional study For RHC; mPAP(mm Hg)/PCWP(mm Hg)/PVR(WU)
1	48	M	55.2	IM	Yes	26.8	71	None/–	92	62	12.5	Sildenafil	NIL, PON, RAD	RHC (–/–/–)
2	46	M	37.1	IM	Yes	51.1	80	None/56.7	81	45	0.0	Sildenafil	NIL, RAD, PON	RHC (57/11/14.5)
3	34	F	32.2	IM, NIL	Yes	22.0	57	None/75	108	21	32.9	Sildenafil, Diuretics	RAD, Asciminib	RHC (42/6/8.9)
4	42	M	71.9	IM	No	70.8	90	None/56	90	49	0.0	Sildenafil	PON, RAD, NIL	chest CT angiography
5	59	F	109.0	IM	Yes	84.8	108	None/63	108	61	0.0	Calcium channel blocker	RAD	chest CT angiography
6	45	F	12.7	IM	Yes	29.5	41	None/58	41	25	0.0	Sildenafil	RAD	–
7	38	F	30.7	IM	No	33.6	46	None/68	91	39	30.5	–	NIL	–
8	72	M	1.6	–	Yes	22.8	47	None/62	47	30	24.6	–	IM, RAD	–
9	35	F	0.4	–	No	35.4	46	None/60	60	34	32.0	–	IM	–
10	57	M	10.4	IM	Yes	12.1	76	None/72	88	51	4.0	–	RAD	–
11	27	F	69.7	IM	No	38.1	95	None/61	95	49	0.1	–	RAD	–
12	33	M	110.2	IM	Yes	155.2	60	None/64	60	45	0.0	–	PON	–
13	57	F	6.3	IM	No	48.2	46	None/67	46	30	0.0	–	RAD	–
14	37	F	63.9	IM, NIL	Yes	35.1	58	None/70	102	28	4.0	Bosentan	RAD	RHC (37/4/11.9)
15	56	F	0.8	–	No	47.6	66	None/65.3	96	39	3.7	–	IM	RHC (40/7/–)
16	58	F	155.5	IM	Yes	12.1	43	None/70	72	54	11.2	–	RAD	–
17	42	F	30.9	IM	Yes	63.0	80	None/68	86	43	2.1	–	NIL	–
18	34	M	0.8	–	No	8.9	44	None/61.1	65	32	33.1	–	IM	–
19	52	M	0.7	–	Yes	40.4	52	None/68	104	43	11.3	Iloprost	NIL	chest CT angiography
20	47	F	0.2	–	Yes	40.8	78	None/56	82	39	0.7	–	RAD	–
21	69	M	223.8	IM	Yes	33.3	52	None/58	52	36	0.0	–	RAD	–
22	63	M	4.4	IM	Yes	4.5	54	None/70.7	62	62	21.9	–	NIL	–
23	36	F	27.8	IM	Yes	28.2	85	None/53.5	108	20	0.0	Sildenafil	PON	–
24	63	M	251.6	IM, RAD	Yes	31.5	51	None/67.6	51	43	0.3	–	–	–
25	68	M	0.1	–	Yes	31.6	86	None/69	86	26	0.0	–	NIL	–
26	54	F	75.7	IM, NIL	Yes	36.3	80	None/44	80	55	0.0	Sildenafil	PON	RHC (41/12/–)
27	54	M	255.6	–	Yes	27.4	117	None/57.4	117	42	4.1	Iloprost	PON	RHC (48/15/–)
28	58	M	0.5	–	Yes	23.5	70	None/71.6	70	69	0.0	–	IM	–
29	33	F	19.9	NIL	Yes	23.2	63	None/56.3	63	51	0.0	–	PON	–

Abbreviations: CML, Chronic Myeloid Leukemia; DAS, Dasatinib; D‐PAH, Dasatinib‐induced Pulmonary Arterial Hypertension; IM, Imatinib; LHD, left heart disease; mPAP, mean pulmonary arterial pressure; NIL, Nilotinib; PCWP, pulmonary capillary wedge pressure; PON, Ponatinib;PVR, pulmonary vascular resistance; RAD, Radotinib; RHC, right heart catheterization; RVSP, Right Ventricular Systolic Pressure; TKI, Tyrosine Kinase Inhibitor.

### 3.6 Outcomes of dasatinib‐induced PAH

After diagnosis of D‐PAH, patients transiently maintained dasatinib with the same dose (*n* = 4), tried to reduce the dose of dasatinib (*n* = 11), or changed immediately to other TKIs (*n* = 14). Ultimately, a total of 29 patients switched to other TKIs (imatinib [*n* = 5], nilotinib [*n* = 7], radotinib [*n* = 10], or ponatinib [*n* = 7]). Sildenafil (*n* = 7), bosentan (*n* = 1), iloprost (*n* = 2), diuretics (*n* = 1), and calcium channel blocker (*n* = 1) were used for D‐PAH treatment (Table 2).

RVSP changes since D‐PAH diagnosis for individual patients are shown in Figure S3. Among 15 patients who maintained dasatinib with the same dose (Patients #1, 17, 18, and 19) or reduced the dose (Patients #3, 7, 8, 9, 10, 14, 15, 16, 20, 22, and 27), 14 patients had experienced RVSP aggravation and switched to other TKIs after a median of 11.3 months (2.1–33.1) from D‐PAH diagnosis. One patient (Patient #8) showed decreased ≤40 mm Hg with the reduced dose of dasatinib and switched to imatinib due to pleural effusion after 24.6 months from D‐PAH diagnosis. At the last follow‐up, 13 patients showed decreased ≤40 mm Hg within a median of 4.5 months (0.9–48.7) from discontinuation of dasatinib, and the other 16 patients remained above 40 mm Hg but showed a gradual decrease except for patient #1. Patient #1 changed from dasatinib to nilotinib at 12.5 months after D‐PAH diagnosis, and RVSP decreased to 44 mm Hg. The patient retried dasatinib due to emergence of E292 V mutation, and it caused RVSP aggravation (63 mm Hg). The patient then switched to ponatinib and later switched to radotinib. During ponatinib and radotinib therapy, his RVSP fluctuated from 46 to 92 mm Hg, and the very last RVSP was elevated to 62 mm Hg. During the median follow‐up of 45.8 months (range, 1.9–148.6 months) from D‐PAH diagnosis, no D‐PAH‐related death occurred.

## 4 DISCUSSION

In this study, we demonstrated that the estimated cumulative incidence of D‐PAH at 3 years was 6.3 ± 1.5%, which appears higher than a study in a French cohort (0.45%)^9^ or a pooled population with Ph^+^ leukemia treated with dasatinib by BMS pharmacovigilance database (0.2%).^10^ However, because these reports counted D‐PAH only confirmed by invasive RHC and the denominators were either all phases of CML or Philadelphia positive leukemia, it is hard to compare these results directly to those of the current study. In the DASISION 3‐year follow‐up report, the results were lower than our study; D‐PAH was reported in eight patients out of 259 CP‐CML patients (3.1%) based on echocardiography, and RHC was performed in only one patient.^23^ One possible explanation was that in our study, among the 679 patients treated with dasatinib therapy, 451 patients who had a little more intentional echocardiography examination at baseline, at pre‐treatment of dasatinib and/or during dasatinib therapy were included as denominators.

PAH has been defined as a resting mean PAP (mPAP) >25 mm Hg or mPAP with exercise >30 mm Hg, the pulmonary arterial wedge pressure ≤15 mm Hg, and a pulmonary vascular resistance >3 Wood unit in the absence of other causes of precapillary PH.^20^ To diagnose PAH, invasive RHC is required, but echocardiography is not recommended and is only used for screening or monitoring.^16^, ^20^ However, in the real world, echocardiography is used for diagnosis more frequently without RHC than with RHC^10^, ^23^. Here, to evaluate the incidence or clinical characteristics of D‐PAH diagnosed by echocardiography, we defined PAH as RVSP >40 mm Hg by echocardiography, which was accepted for the diagnosis of PAH with relevant symptoms and the absence of other specific causes.^24^, ^25^, ^26^, ^27^ RVSP >40 mm Hg was detected in 56 patients during dasatinib therapy. Among the 56 patients, 27 were ruled out for D‐PAH because they were asymptomatic or showed symptoms due to other causes. During continuation of dasatinib, no one developed D‐PAH with continuous dasatinib treatment. Thus, for asymptomatic patients showing RVSP >40 mm Hg once, immediate dasatinib discontinuation may not be necessary, but periodic echocardiography is needed. In addition, five patients with RVSP elevation more than 40 mm Hg at baseline were treated with/without prior TKIs (imatinib [*n* = 2], radotinib [*n* = 1], imatinib and radotinib [*n* = 1], naïve [*n* = 1]). These patients did not develop further symptoms and in three patients with follow‐up echocardiography, elevated RVSP was normalized regardless of continuing dasatinib treatment.

Interestingly, we found a trend that RVSP increased over time. The mean RVSP measured during dasatinib was significantly increased after taking dasatinib compared with baseline. However, this result should be interpreted cautiously because this may be reflecting the late diagnosis of D‐PAH by the nonperiodic echocardiographic screening. This is the first report to examine the serial dynamic change of RVSP by echocardiography. Our results suggest that if a patient develops dyspnea with long‐term dasatinib treatment, echocardiography examination may provide a benefit for early diagnosis of D‐PAH. Follow‐up echocardiography may be warranted for patients with long‐term dasatinib treatment.

In terms of clinical features of D‐PAH, in this study, D‐PAH was diagnosed at a median of 39.2 months (16.1–155.2) after dasatinib initiation, which was consistent with previous reports; however, we did not found female predominance, which was a discordant finding to previous reports.^9^, ^10^ In the comparison between asymptomatic and D‐PAH groups in patients with RVSP >40 mm Hg, the D‐PAH group showed a trend toward higher mean daily dose and higher concurrent pleural effusions. The concurrent pleural effusions were observed in 75.9% of patients with D‐PAH, which was slightly higher than 68% in previous reports.^9^, ^10^ Although Src inhibition‐mediated vasoconstriction may play an important role in D‐PAH, Guignabert et al. demonstrated that dasatinib causes pulmonary arterial endothelial cell dysfunction and remodeling, suggesting a second‐hit was necessary to cause overt PAH in a susceptible individual.^12^, ^28^ Dasatinib dosing and pleural effusion may be also related to the degree of endothelial dysfunction.

To evaluate the long‐term outcomes of D‐PAH, we followed the symptoms and RVSP using echocardiography for 29 patients with D‐PAH. After D‐PAH diagnosis, patients who tried the same or reduced dose of dasatinib experienced RVSP aggravation, except for one patient, and ultimately switched to other TKIs. This suggested that dasatinib treatment should be promptly halted with a diagnosis of D‐PAH. After dasatinib discontinuation, clinical symptoms and RVSP were normalized in 13 patients (45%) and 15 patients (52%) showed a gradual decrease above 40 mm Hg. One patient, who resolved after changing to nilotinib, retried dasatinib due to emergence of E292V mutation and experienced again RVSP elevation, suggesting that physicians should be very cautious when considering reintroduction of dasatinib even in unavoidable situations. Overall, our result of long‐term resolution with the median follow‐up of 45.8 months from D‐PAH diagnosis is comparable with a previous report that showed 94% of improvement including 58% of complete resolution.^10^ Although D‐PAH has a reversible feature, which is rarely observed in other types of PAH that are almost progressive and irreversible,^1^, ^29^ close surveillance using echocardiography and relevant symptoms are warranted. The current guidelines recommend RHC to confirm the diagnosis of PAH and support a treatment decision.^20^ Really, among the patients with suspected D‐PAH by only echocardiography, some patients may have other etiologies such as coronary artery disease, pulmonary thromboembolism, and connective tissue disease, and thus for the selected patients, RHC should be recommended.

We acknowledge several limitations of our study, such as its nonprospective design and various intervals of echocardiography. However, we followed up the long‐term clinical and hemodynamic changes using echocardiography in a large number of patients treated with dasatinib and demonstrated that for a patient who had RVSP >40 mm Hg with relevant clinical manifestations, non‐invasive echocardiography can be fast way for early diagnosis as well as for subsequent monitoring of D‐PAH. Moreover early discontinuation of dasatinib will be helpful for complete resolution. In addition, for a small number of patients who showed RVSP >40 mm Hg without relevant symptoms, periodic RVSP monitoring can be recommended without dasatinib discontinuation.

## CONFLICTS OF INTEREST

The authors declare no conflict of interest.

## AUTHOR CONTRIBUTIONS

D‐WK had primary responsibility for study design, collection and assembly of data, data analysis and interpretation, and manuscript writing. S‐EL and JHK performed statistical analysis and interpreted data, and wrote the manuscript. N‐GC, BC, and E‐JJ contributed to data collection. H‐EJ, H‐JY, and W‐BC performed echocardiography and interpreted data.

## Supporting information

Supplementary Material

## Data Availability

The data of the current study are available from the corresponding author on a reasonable request.
