# Supplementary material for: Change of right ventricular systolic pressure can indicate dasatinib‐induced pulmonary arterial hypertension in chronic myeloid leukemia
Source: Cancer Med. 2021 Feb 15;10(5):1515–24. doi: 10.1002/cam4.3588 (PMC7940235; doi:10.1002/cam4.3588)
Supplement: Supplementary file 1 — Supplementary Material [file CAM4-10-1515-s001.docx]

**Supplement Table 1. Clinical Characteristics of patients with RVSP >40mmHg**

|  | D-PAH  (n=29) | Asymptomatic  (n=27) | *p* |
| --- | --- | --- | --- |
| Age-das, years, median (range) | 46 (21–70) | 61 (26–75) | <0.001 |
| Sex, male, n (%) | 14 (48.3) | 12 (44.4) | 0.774 |
| Dasatinib frontline n (%) | 9 (31.0) | 11 (40.7) | 0.449 |
| Previous treatment |  |  |  |
| Interferon, n (%) | 7 (24.1) | 2 (7.4) | 0.146 |
| Allogeneic HSCT, n (%) | 1 (3.4) | 0 (0.0) | 1.000 |
| Prior TKI to dasatinib |  |  |  |
| Imatinib, n (%) | 19 (65.5) | 14 (51.9) | 0.299 |
| Nilotinib, n (%) | 4 (13.8) | 5 (18.5) | 0.725 |
| Bosutinib, n (%) | 0 (0.0) | 0 (0.0) | - |
| Radotinib, n (%) | 1 (3.4) | 4 (14.8) | 0.185 |
| Ponatinib, n (%) | 0 (0.0) | 0 (0.0) | - |
| Time from CML diagnosis to dasatinib initiation, months, median (range) | 30.7 (0.1–255.6) | 4.2 (0.3–174.6) | 0.071 |
| Time from dasatinib initiation to RVSP >40 mmHg, months, median (range) | 39.2 (16.1–155.2) | 8.8 (0.3–92.9) | <0.001 |
| Follow up duration after dasatinib, months, median (range) | 39.3(16.1–155.2) | 25.4(2.4–148.2) | 0.003 |
| Mean daily dose of dasatinib, mg/day, median (range) | 95 (54–132) | 77 (56–140) | 0.089 |
| Pleural effusion, n (%) | 22 (75.9%) | 14 (51.9%) | 0.061 |
| Median highest RVSP, mmHg (range) | 82 (41–117) | 43 (41–58) | <0.001 |

**Abbreviations:** CML, Chronic Myeloid Leukemia; D-PAH, Dasatinib-induced Pulmonary Arterial Hypertension; HSCT, Hematopoietic Stem Cell Transplantation; RVSP, Right Ventricular Systolic Pressure; TKI, Tyrosine Kinase Inhibitor

**Supplementary Figure legends**

**Supplement Figure 1. RVSP change according to frontline and subsequent line dasatinib therapies.** A total of 196 patients (43.5%) received first-line dasatinib therapy and 255 (56.5%) patients received dasatinib as a subline therapy after prior TKI. RVSPs of frontline and subsequent line dasatinib treatment groups were plotted according to implemented time and 1-year interval from dasatinib initiation (A and B). RVSP changes in both groups were compared (C).

**Supplement Figure 2. Change of RVSP since the date of RVSP >40 mmHg in the asymptomatic group.**

**Supplement Figure 3. Change of RVSP since the diagnosis of D-PAH.**

**
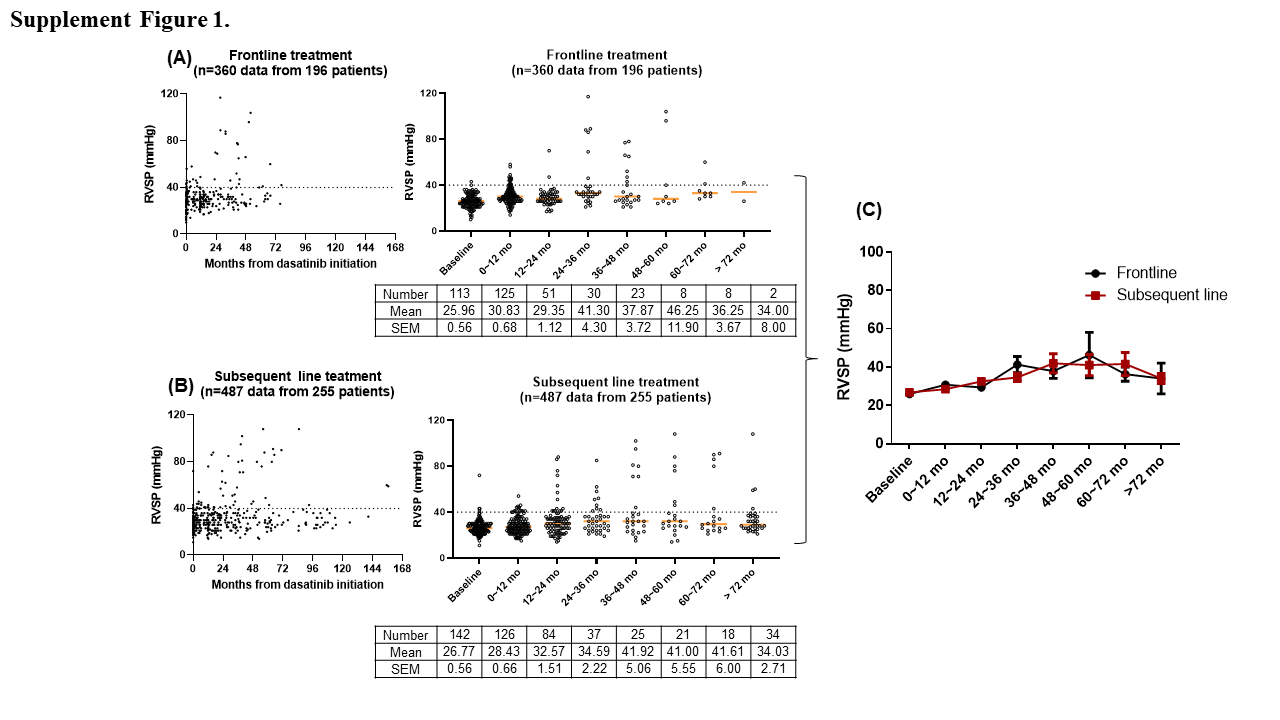
**

**
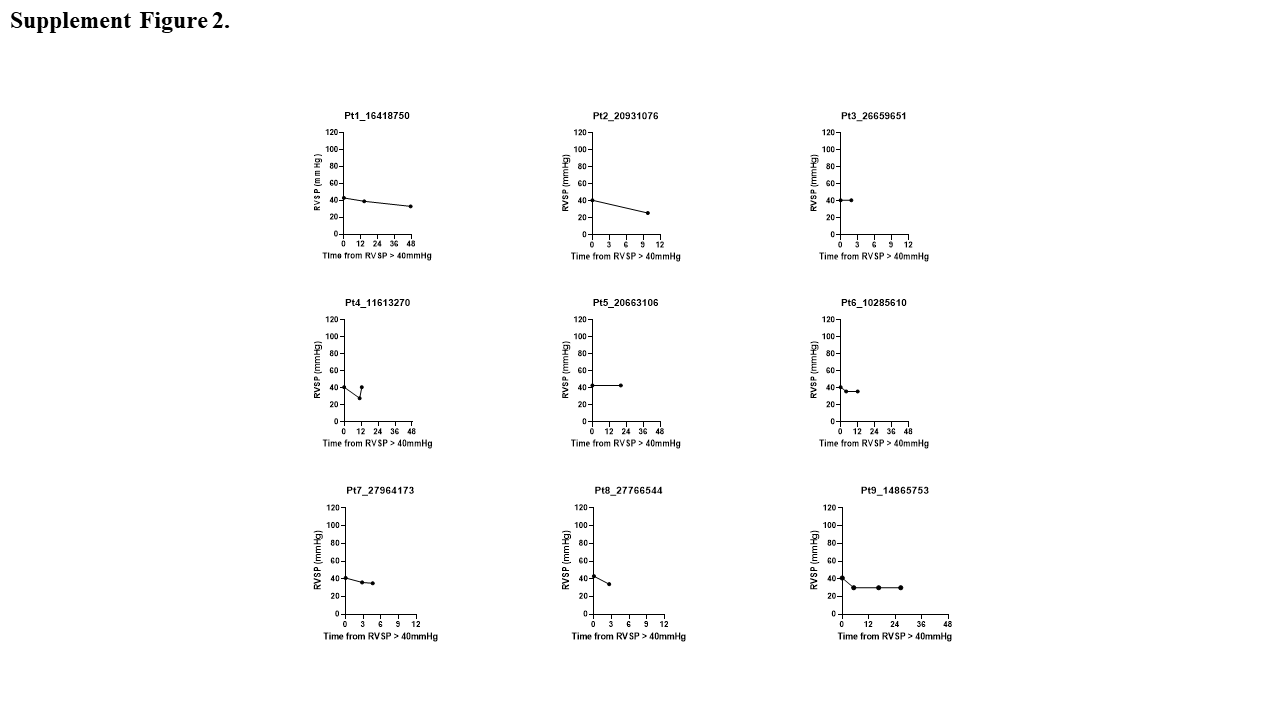
**

**
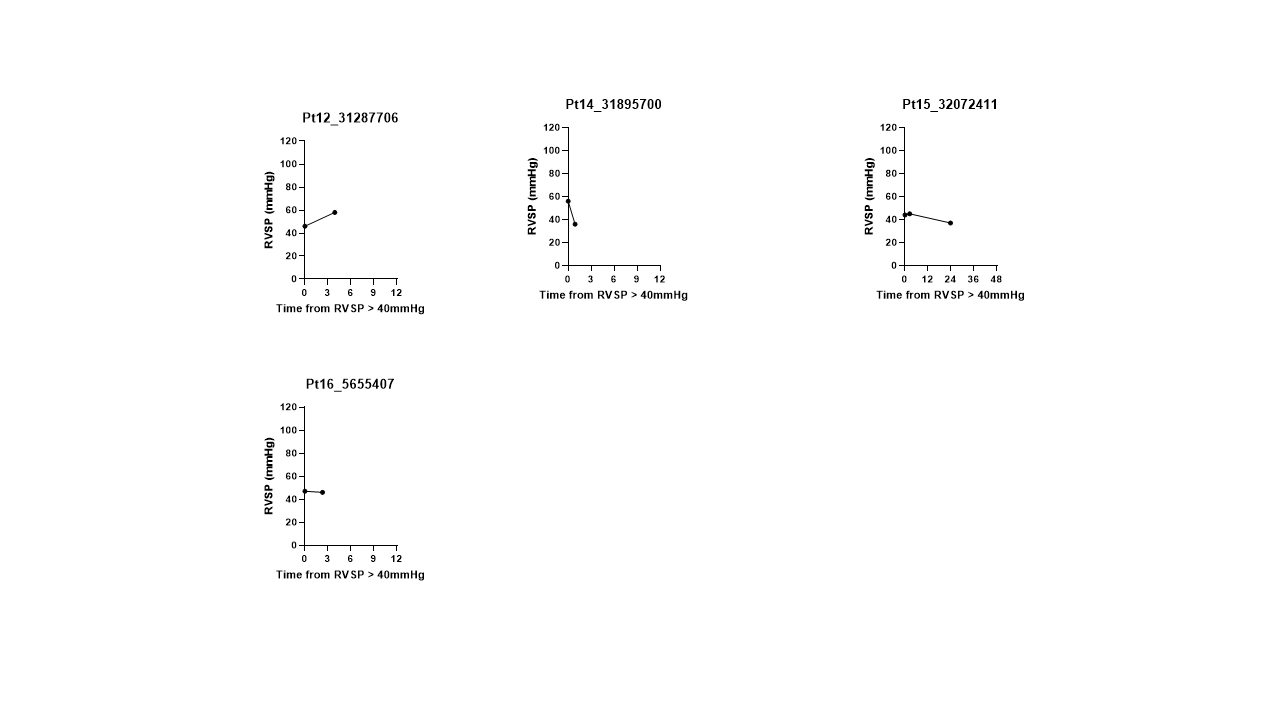
**

**
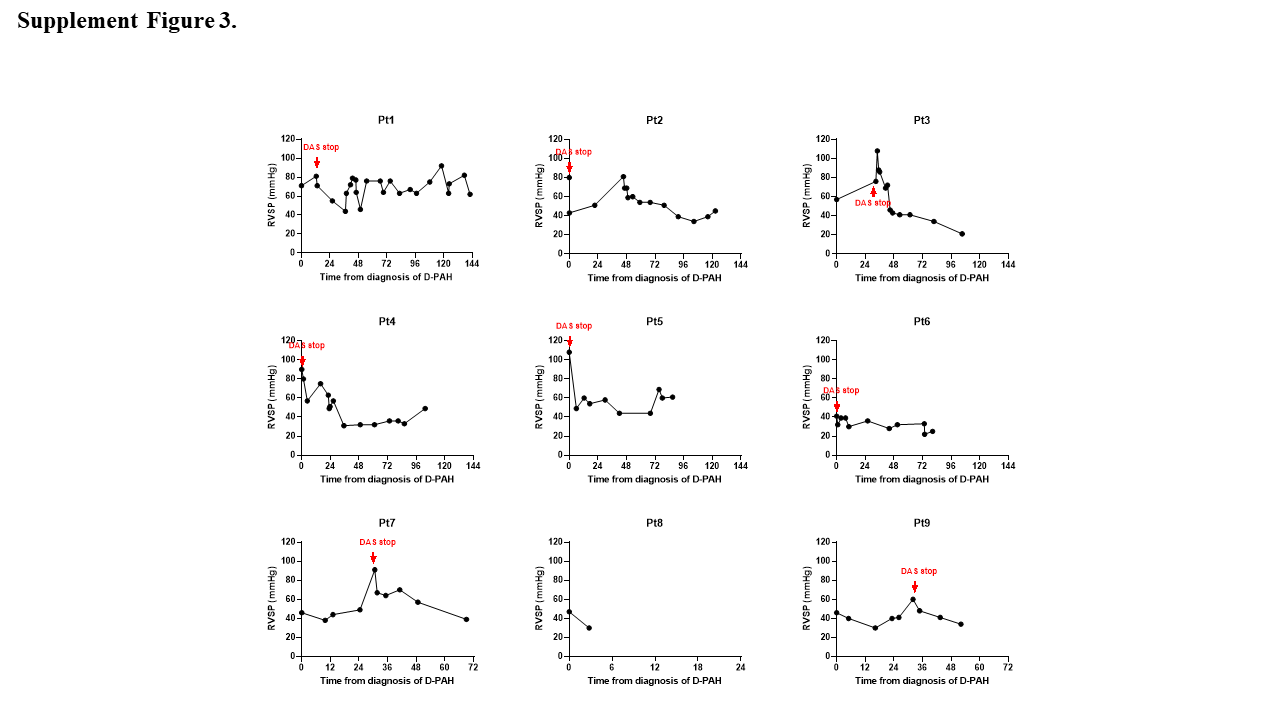
**

**
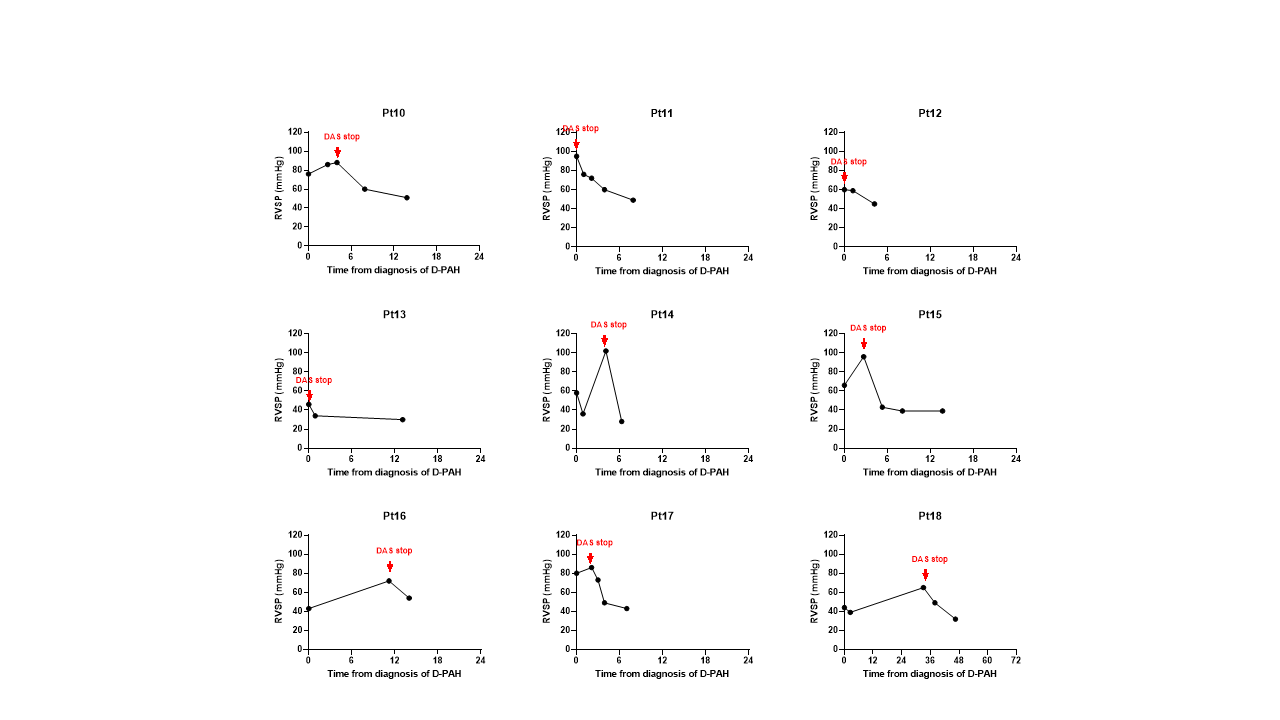
**

**
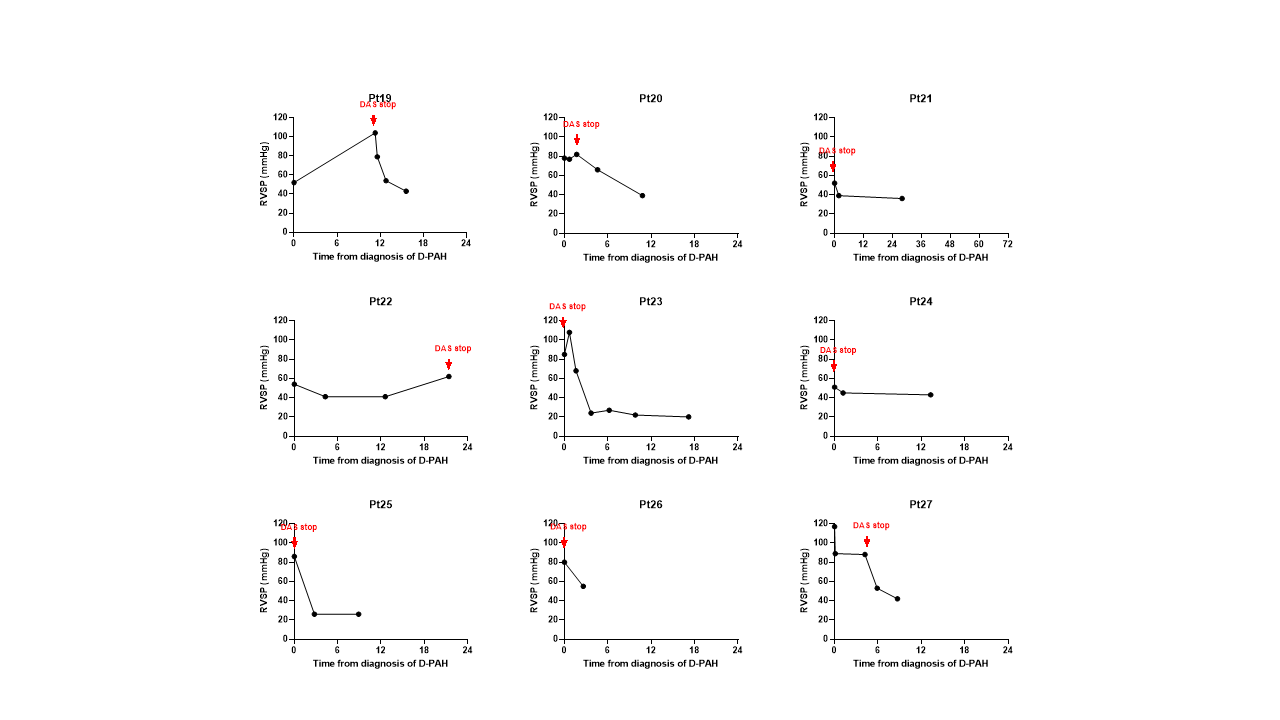
**

**
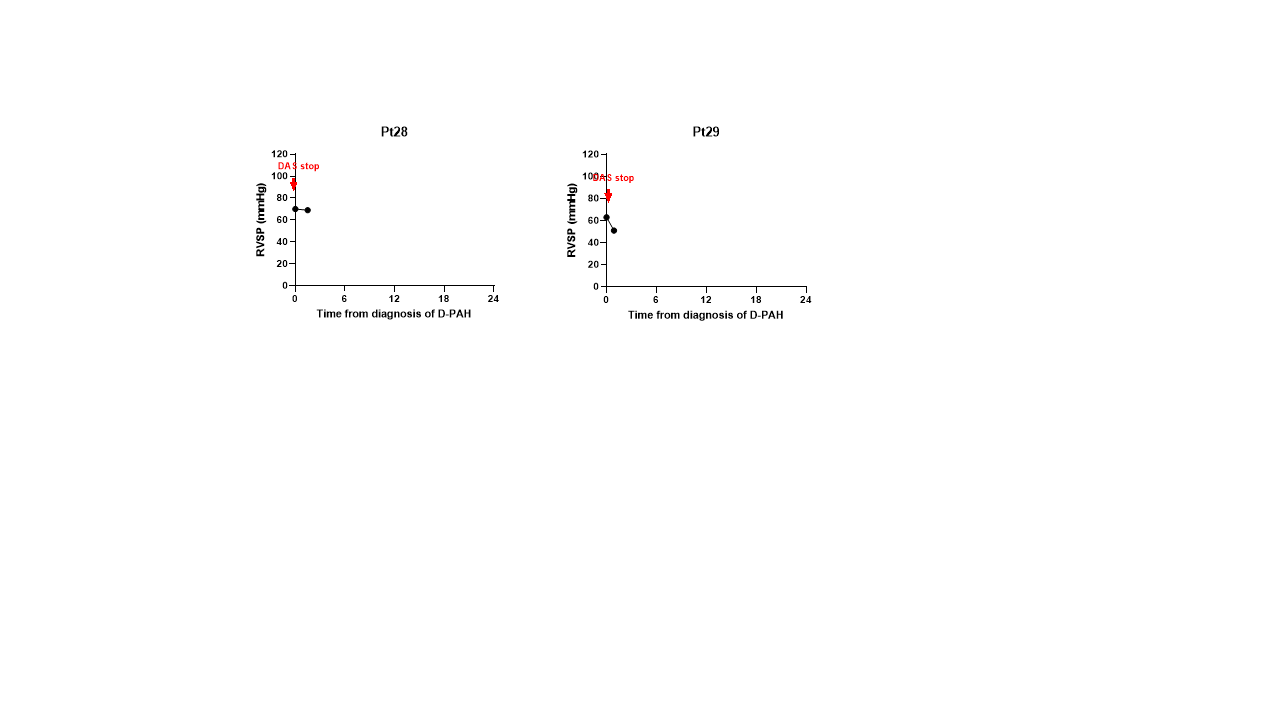
**
